# Causal discovery and inference: concepts and recent methodological advances

**DOI:** 10.1186/s40535-016-0018-x

**Published:** 2016-02-18

**Authors:** Peter Spirtes, Kun Zhang

**Affiliations:** Department of Philosophy, Carnegie Mellon University, Pittsburgh, USA; Max-Planck Institute for Intelligent Systems, 72076 Tübingen, Germany

**Keywords:** Causal inference, Causal discovery, Structural equation model, Conditional independence, Statistical independence, Identifiability

## Abstract

This paper aims to give a broad coverage of central concepts and principles involved in automated causal inference and emerging approaches to causal discovery from i.i.d data and from time series. After reviewing concepts including manipulations, causal models, sample predictive modeling, causal predictive modeling, and structural equation models, we present the constraint-based approach to causal discovery, which relies on the conditional independence relationships in the data, and discuss the assumptions underlying its validity. We then focus on causal discovery based on structural equations models, in which a key issue is the identifiability of the causal structure implied by appropriately defined structural equation models: in the two-variable case, under what conditions (and why) is the causal direction between the two variables identifiable? We show that the independence between the error term and causes, together with appropriate structural constraints on the structural equation, makes it possible. Next, we report some recent advances in causal discovery from time series. Assuming that the causal relations are linear with nonGaussian noise, we mention two problems which are traditionally difficult to solve, namely causal discovery from subsampled data and that in the presence of confounding time series. Finally, we list a number of open questions in the field of causal discovery and inference.

## Background

The goal of many sciences is to understand the mechanisms by which variables came to take on the values they have (i.e., to find a generative model), and to predict what the values of those variables would be if the naturally occurring mechanisms in a population^1^ were subject to outside *manipulations*. For example, a *randomized experiment* is one kind of manipulation, which substitutes the outcome of a randomizing device to set the value of a variable, such as whether or not a particular diet is used, instead of the naturally occurring mechanism that determines diet. In nonexperimental settings, biologists gather data about the gene activation levels in normally operating systems, and seek to understand which genes affect the activation levels of which other genes and seek to predict what the effects of intervening to turn some genes on or off would be; epidemiologists gather data about dietary habits and life expectancy in the general population and seek to find what dietary factors affect life expectancy and to predict the effects of advising people to change their diets. Finding answers to questions about the mechanisms by which variables come to take on values, or predicting the value of a variable after some other variable has been manipulated, is characteristic of causal inference. If only observational (nonexperimental) data are available, predicting the effects of manipulations typically involves drawing samples from one density (of the unmanipulated population) and making inferences about the values of a variable in a population that has a different density (of the manipulation population).

Many of the basic problems and basic assumptions remain the same across domains. In addition, although there are some superficial similarities between traditional supervised machine learning problems and causal inference (e.g., both employ model search and feature selection, the kinds of models employed overlap, and some model scores can be used for both purposes), these similarities can mask some very important differences between the two kinds of problems.

### History

Traditionally, there have been a number of different approaches to causal discovery. The gold standard of causal discovery has typically been to perform planned or randomized experiments (Fisher 1970). There are obvious practical and ethical considerations that limit the application of randomized experiments in many instances, particularly on human beings. Moreover, recent data collection techniques and causal inference problems raise several practical difficulties regarding the number of experiments that need to be performed in order to answer all of the outstanding questions (Eberhardt et al. 2005, 2006).

## Manipulating and conditioning

Conditioning maps a given joint density, and a given subpopulation (typically specified by a set of values for random variables) into a new density. The conditional density is a function of the joint density over the random variables and a set of values for a set of random variables.^2^ The estimation of a conditional probability is often nontrivial because the number of measurements in which the variables conditioned on that take on a particular value might be small. A large part of statistics and machine learning is devoted to estimating conditional probabilities from realistic sample sizes under a variety of assumptions.

More generally, suppose the goal is to find a “good” predictor of the value of some target variable *Y* from the values of the observed covariates $\mathbf {O}$, for a unit. We will refer to this as Problem 1, described more formally below. Ultimately, the prediction of the value of *Y* is performed by some prediction function $\hat{Y}_n(\mathbf {O})$. One traditional measure of how good the predictor $\hat{Y}_n(\mathbf {O})$ is in predicting *Y* is the mean squared prediction error (MSPE), which is equal to $E[(Y - \hat{Y}_n(\mathbf {O}))^2]$, where the expected value is taken with respect to the density $p(\mathbf {O},Y)$ (Bickel and Doksum 2000).^3^
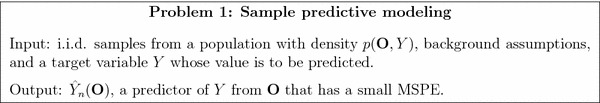


In addition to predicting future values of random variables from the present and past values, conditional probabilities are also useful for predicting hidden values at the current time.

### Manipulated probabilities

A *manipulated* density results from taking action on a given population—it may or may not be equal to any observational conditional density, depending upon what the causal relations between variables are. Manipulated probability densities are the appropriate probability densities to use when making predictions about the effects of taking actions (“manipulating” or “doing”) on a given population (e.g., assigning satellite readings), rather than observing (“seeing”) the values of given variables. A manipulation *M* specifies a new conditional probability density for some set of variables. If $\mathbf {X}$ and $\mathbf {O}$ are sets of variables with density $p(\mathbf {X}|\mathbf {O})$, a manipulation *M* changes the density to some new density $p'(\mathbf {X}|\mathbf {O})$. Manipulated probabilities are the probabilities that are implicitly used in decision theory, where the different actions under consideration are manipulations.^4^ We designate the density of a set of variables $\mathbf {V}$ after a manipulation *M* as $p(\mathbf {V}||M)$. Each manipulation is assumed to be an ideal manipulation in the following senses:

1. Each manipulation succeeds, i.e., if the manipulation is designated as setting the density to $p'(\mathbf {X}|\mathbf {O})$, then the post-manipulation density is $p'(\mathbf {X}|\mathbf {O})$.
2. There is no fat hand, i.e., each manipulation directly affects only the variables manipulated.

A probability model specifies a density over a set of random variables $\mathbf {O}$. A causal model specifies a set of densities over a set of random variables $\mathbf {O}$, one for each possible manipulation *M* of the random variables in $\mathbf {O}$, including the null manipulation. Hence, a probability model is a member of a causal model.

Given a set of variables $\mathbf {V}$, the direct causal relations among the variables can be represented by a directed graph, where the variables in $\mathbf {V}$ are the vertices, and there is an edge from *A* to *B* if *A* is a direct cause of *B* relative to $\mathbf {V}$.

We will refer to the problem of estimating manipulated densities given a sample from a marginal unmanipulated density, a (possibly empty) set of samples from manipulated densities, and background assumptions, as Problem 2; it is stated more formally below. In contrast to conditional probabilities, which can be estimated from samples from a population, typically the gold standard for estimating manipulated densities is an experiment, often a randomized trial. However, in many cases, experiments are too expensive, too difficult, or not ethical to carry out. This raises the question of what can be determined about manipulated probability densities from samples from a population, possibly in combination with a limited number of randomized trials. The problem is even more difficult because the inference is made from a set of measured random variables $\mathbf {O}$ from samples that might not contain variables that are causes of multiple variables in $\mathbf {O}$.

Problem 2 is usually broken into two parts: finding a set of causal models from sample data, some manipulations (experiments) and background assumptions, and predicting the effects of a manipulation given a causal model. Here, a “causal model” (Sect. 3) specifies for each possible manipulation that can be performed on the population (including the manipulation that does nothing to a population) a post-manipulation density over a given set of variables.
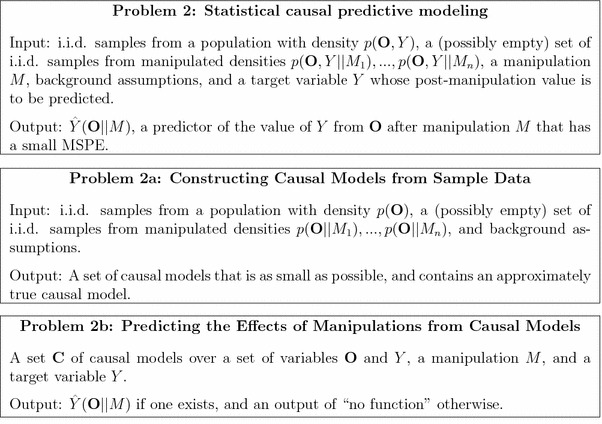


The reason why the stated goal for the output of Problem 2a is a set of causal models, rather than a single causal model, is that in some cases it is not possible to reliably find a true causal model given the inputs. Furthermore, in contrast to predictive models, even if a true causal model can be inferred from a sample from the unmanipulated population, it generally cannot be validated on a sample from the unmanipulated population, because a causal model contains predictions about a manipulated population that might not actually exist. This has been a serious impediment to the improvement of algorithms for constructing causal models, because it makes evaluating the performance of such algorithms difficult. It is possible to evaluate causal inference algorithms on simulated data, to employ background knowledge to check the performance of algorithms, and to conduct limited (due to expense, time, and ethical constraints) experiments, but these serve as only partial checks how algorithms perform on real data in a wide variety of domains.

## Structural equation models

The set of random variables in a structural equation model (SEM) can be divided into two subsets, the “error variables” or “error terms,” and the substantive variables (for which there is not standard terminology in the literature). The substantive variables are the variables of interest, but they are not necessarily all observed. Each substantive variable *X* is a function of other substantive variables $\mathbf {V}$, and a unique error term ${\varepsilon _X}$, i.e., $X := f(\mathbf {V}, \varepsilon _X)$. We use an assignment operator, rather than an equality operator because the equations are interpreted causally; manipulating a variable in $\mathbf {V}$ can lead to a change in the value of *X*.

**Fig. 1:**
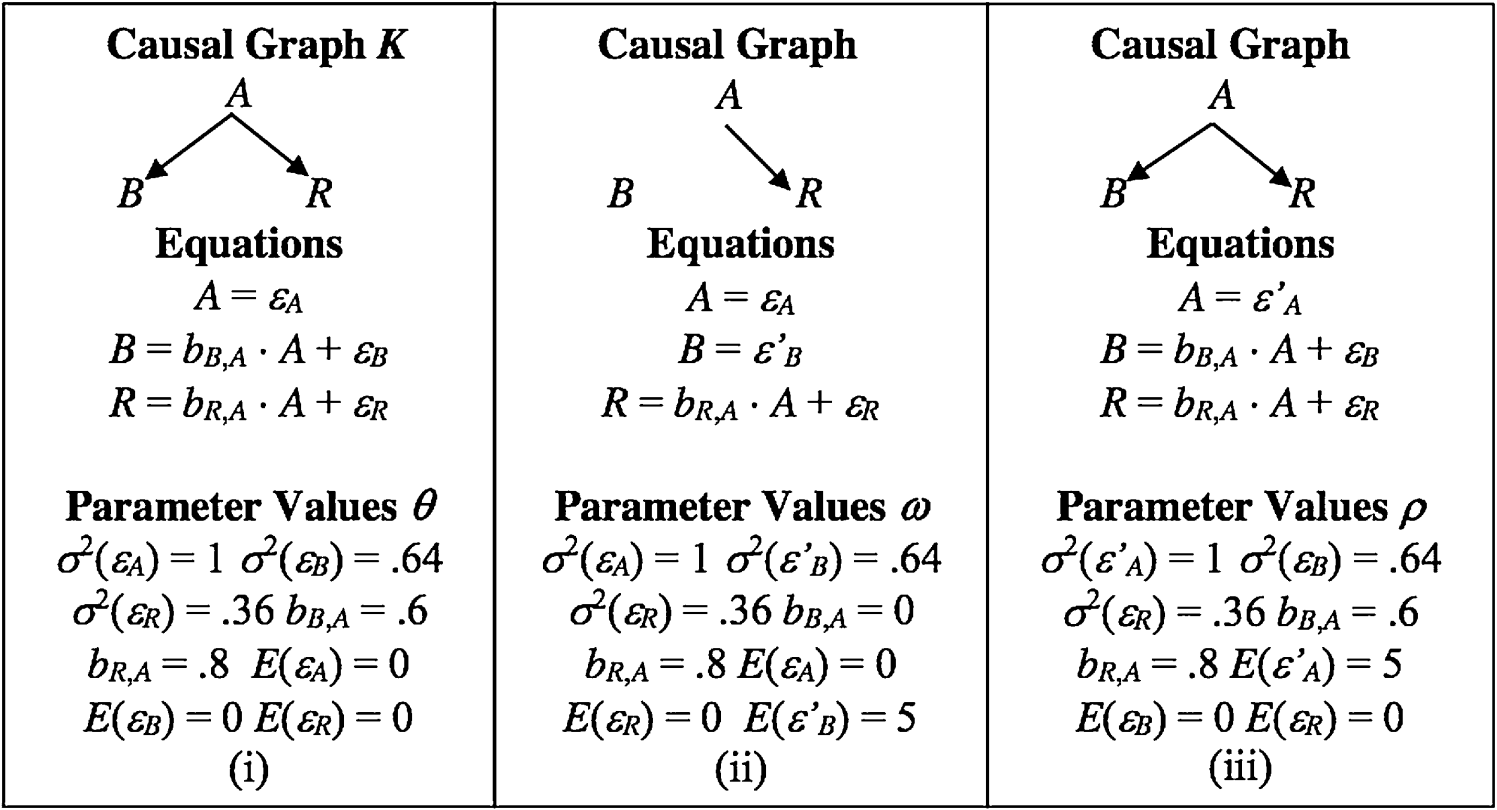
**a** Unmanipulated causal graph *K*; **b** *B* Manipulated to 5; **c** *A* Manipulated to 5

Each SEM is associated with a directed graph whose vertices include the substantive variables, and that represents both the causal structure of the model and the form of the structural equations. There is a directed edge from *A* to *B* ($A\rightarrow B$) if the coefficient of *A* in the structural equation for *B* is nonzero. In a linear SEM, the coefficient $b_{B,A}$ of *A* in the structural equation for *B* is the *structural coefficient associated* with the edge $A \rightarrow B$. In general, the graph of a SEM may have cycles (i.e., directed paths from a variable to itself) and may explicitly include error terms with double-headed arrows between them to represent that the error terms are dependent (e.g., $\varepsilon _A \leftrightarrow \varepsilon _B$); if no such edge exists in the graph, the error terms are assumed to be independent. If a variable has no arrow directed into it, then it is *exogenous*; otherwise, it is *endogenous*. In SEM $K(\varvec{\theta })$ depicted in Fig. 1a (where $\varvec{\theta }$ is the set of parameter values for *K*), *A* is exogenous and *B* and *R* are endogenous. If the graph has no directed cycles and no double-headed arrows, then it is a *directed acyclic graph* (DAG).

Given the independent error terms in SEM *K*, for each $\varvec{\theta }$, SEM *K* entails both a set of conditional independence relations among the substantive variables, and that the joint density over the substantive variables *factors according to the graph*, i.e., the joint density can be expressed as the product of the density of each variable conditional on its parents in the graph. For example, $p(A,B,R) = p(A)p(B|A)p(R|A)$ for all $\varvec{\theta }$. This factorization in turn is equivalent to a set of conditional independence relations among the substantive variables (Lauritzen et al. 1990).

$I_p({\mathbf {X}},{\mathbf {Y}}|{\mathbf {Z}})$ denotes that ${\mathbf {X}}$ is independent of ${\mathbf {Y}}$ conditional on ${\mathbf {Z}}$ in density *p*, i.e., $p({\mathbf {X}}|{\mathbf {Y}},{\mathbf {Z}}) = p({\mathbf {X}}|{\mathbf {Z}})$ for all $p({\mathbf {X}}|{\mathbf {Z}}) \ne 0$. (In cases where it does not create any ambiguity, the subscript *p* will be dropped.) If a SEM *M* with parameter values $\varvec{\theta }$ (represented by $M(\varvec{\theta })$) entails that ${\mathbf {X}}$ is independent of ${\mathbf {Y}}$ conditional on ${\mathbf {Z}}$, we write $I_{M(\varvec{\theta })}({\mathbf {X}},{\mathbf {Y}}|{\mathbf {Z}})$. If a SEM with fixed causal graph *M* entails that $I_{M({\varvec{\Theta }})}({\mathbf {X}},{\mathbf {Y}}|{\mathbf {Z}})$ for all possible parameter values $\varvec{\Theta },$ we write $I_{M}({\mathbf {X}},{\mathbf {Y}}|{\mathbf {Z}})$. In that case we say that *M* entails $I({\mathbf {X}},{\mathbf {Y}}|{\mathbf {Z}})$. It is possible to determine whether $I_{M}({\mathbf {X}},{\mathbf {Y}}|{\mathbf {Z}})$ from the graph of *M* using the purely graphical criterion, “d-separation” (Pearl 1988).

A Bayesian network is a pair $\langle G, p \rangle$, where *G* is a DAG and a *p* is a probability density such that if ${\mathbf {X}}$ and ${\mathbf {Y}}$ are d-separated conditional on ${\mathbf {Z}}$ in *G*, then ${\mathbf {X}}$ and ${\mathbf {Y}}$ are independent conditional on ${\mathbf {Z}}$ in *G*. If the error terms in a SEM with a DAG *G* are jointly independent, and $p(\mathbf {V})$ is the entailed density over the substantive variables, then $\langle G, p(\mathbf {V}) \rangle$ is a Bayesian network.

### Representing manipulations in a SEM

Given a linear SEM, a manipulation of a variable $X_i$ in a population can be described by the following kind of equation: $X_i = \sum _{X_j \in \mathbf {PA}(X_i)} b_{i,j} X_j + \varepsilon _i$, where all of the variables are the post-manipulation variables, $\mathbf {PA}(X_i)$ is a new set of causes of $X_i$ (which are included in the set of noneffects of $X_i$ in the unmanipulated population). A simple special case is where $X_i$ is set to a constant *c*.

In a causal model such as SEM $K(\varvec{\theta })$, the post-manipulation population is represented in the following way, as shown in Fig. 1. The result of modifying the set of structural equations in this way can lead to a density in the randomized population that is not necessarily the same as the density in any subpopulation of the general population. [For more details see Pearl (2000); Spirtes et al. (2001)]. See Fig. 1 for the examples of manipulations to SEM *K*.

A set $\mathbf {S}$ of variables is *causally sufficient* if every variable *H* that is a direct cause (relative to $\mathbf {S }\cup \{H\}$) of any pair of variables in $\mathbf {S}$ is also in $\mathbf {S}$. Intuitively, a set of variables $\mathbf {S}$ is causally sufficient if no common direct causes (relative to $\mathbf {S}$) have been left out of $\mathbf {S}$. If SEM *K* is true, then $\{A,B,R\}$ is causally sufficient, but $\{B,R\}$ is not because *A* is a common direct cause of *B* and *R* relative to $\{A,B,R\}$ but is not in $\{B,R\}$. If the observed set of variables is not causally sufficient, then the causal model is said to contain *unobserved common causes*, *hidden common causes*, or *latent variables*.

## Assumptions

The following assumptions are often used to relate causal relations to probability densities.

### The causal Markov assumption

#### Causal Markov assumption

 For causally sufficient sets of variables, all variables are independent of their noneffects (nondescendants in the causal graph) conditional on their direct causes (parents in the causal graph) (Spirtes et al. 2001).

The causal Markov assumption is an oversimplification because it basically assumes that all associations between variables are due to causal relations. There are several other ways that associations can be produced.

First, conditioning on a common descendant can produce a conditional dependency. For example, if sex and intelligence are unassociated in the population, but only the most intelligent women attend graduate school, while men with a wider range of intelligence attend graduate school, then sex and intelligence will be associated in a sample consisting of graduate students (i.e., sex and intelligence cause graduate school attendance, which has been conditioned on in the sample). See Spirtes et al. (1995) for a discussion of selection bias. Second, logical relationships between variables can also produce noncausal correlations (e.g., if $GDP\_{yearly}$ is defined to be the sum of $GDP\_{January}$, $GDP\_{February}$, etc., $GDP\_{yearly}$ will be associated with these variables, but not caused by them). For a discussion of logical relations between variables, see Spirtes and Scheines (2004). Third, it does not have any way of dealing with instantaneous symmetric interactions (like classical theories of gravity).

### The causal faithfulness assumption

**Fig. 2:**
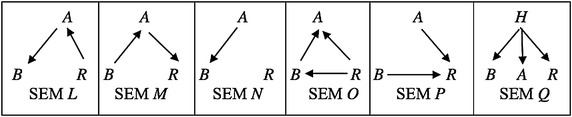
Alternative SEM models

Consider SEM *O* in Fig. 2. Suppose we have $I_K(B,R|A)$, where SEM *K* is shown in Fig. 1a, whereas it is not the case that $I_O(B,R|A)$. However, just because *O* does not entail $I_O(B,R|A)$ for all sets of parameter values $\varvec{\beta }$, that does not imply that there are *no*$\varvec{\beta }$ for which $I_{O(\varvec{\beta })})(B,R|A)$. For example, if the variances of *R*, *A*, and *B* are all 1, for any $\varvec{\beta }$ for which $\text {cov}_{O(\varvec{\beta })}(A,B)\cdot \text {cov}_{O(\varvec{\beta })}(A,R) = \text {cov}_{O(\varvec{\beta })}(B,R)$, it follows that $\text {cov}_{O(\varvec{\beta })}(B,R|A) = 0$. This occurs when $(b_{B,R} \cdot b_{A,R} + b_{A,B}) \cdot (b_{B,R} \cdot b_{A,B} + b_{A,R}) = b_{R,B}$. So if $I_p(B,R|A)$ is true in the population, there are at least two kinds of explanation: any set of parameter values for SEMs *K* (in Fig. 1a), *L*, or *M* (in Fig. 2), on the one hand, or any parameterization of SEM *O* for which $(b_{B,R} \cdot b_{A,R} + b_{A,B}) \cdot (b_{B,R} \cdot b_{A,B} + b_{A,R}) = b_{R,B}$. There are several arguments why, although *O* with the special parameter values is a possible explanation, in the absence of evidence to the contrary, *K*, *L*, or *M* should be the preferred explanations.

First, *K*, *L*, and *M* explain the independence of *B* and *R* conditional on *A* structurally, as a consequence of no direct causal connection between the variables. In contrast *O* explains the independence as a consequence of a large direct effect of *B* on *R* canceled *exactly* by the product of large direct and indirect effects of *B* and *R* on *A*.

Second, this cancelation is improbable (in the Bayesian sense that if a zero conditional covariance is not entailed, the measure of the set of free parameter values for any DAG that lead to such cancelations is zero for any “smooth” prior probability density,^5^ such as the Gaussian or exponential one, over the free parameters).

Finally, *K*, *L*, and *M* are simpler than *O*. *K*, *L*, and *M* have fewer free parameters than *O*.

The assumption that a causal influence is not hidden by coincidental cancelations can be expressed for SEMs in the following way: A density *p* is *faithful* to the graph *G* of a SEM if and only if every conditional independence relation true in *p* is entailed by *G*.

#### Causal faithfulness assumption

For a causally sufficient set of variables $\mathbf {V}$ in a population *P*, the population density $p_P(\mathbf {V})$ is faithful to the causal graph over $\mathbf {V}$ for *P* (Spirtes et al. 2001).

The causal faithfulness assumption requires preferring *K*, *L*, and *M* to *O*, because parameter values $\varvec{\beta }$ for which $I_{O(\varvec{\beta })})(B,R|A)$ would violate the Causal Faithfulness Assumption. Recently, there have been a number of search algorithms that are consistent, but have substituted other kinds of assumptions in place of the causal faithfulness assumption.

### The output of a search for causal models

The following sections describe different possible alternatives that can be output by a reliable search algorithm.

#### Markov equivalence classes

A trek between *A* and *B* is either a directed path from *A* to *B*, a directed path from *B* to *A*, or a path between *A* and *B* that does not contain a subpath $X \rightarrow Y \leftarrow Z$. SEMs *K*, *L*, and *M* are Markov equivalent, in the sense that their respective graphs all entail the same set of conditional independence relations. If *K* is true, any SEM with a graph that contains no path between *A* and *R* can be eliminated from consideration by the causal Markov assumption (e.g., *N* in Fig. 2). SEM *P* also violates the Causal Markov Assumption. *O* is incompatible with the population conditional independencies by the causal faithfulness assumption. However, neither of these assumptions implies *L* or *M* is incompatible with the population conditional independencies.

Since *K*, *L*, and *M* entail the same set of conditional independence relations, it is not possible to eliminate *L* or *M* as incompatible with the population conditional independence relations without either adding more assumptions or background knowledge or using features of the probability density that are not conditional independence relations. In the case of linear SEMs with Gaussian error terms (and for multinomial Bayesian networks), there are no other features of the density that distinguish *K* from *L* or *M*. However, as we will illustrate later, for other families of distributions, there are nonconditional independence constraints that can be entailed by a graph that do distinguish *K* from *L* or *M*.

#### Distribution equivalence

*K* and *L* are *distribution equivalent* if and only if for any assignment of parameter values $\varvec{\theta }$ to *K* there exists an assignment of parameter values $\varvec{\theta }'$ to *L* that represents the same density, and vice versa. If all of the error terms are Gaussian with linear causal relations, then *K* and *L* are distribution equivalent as well as Markov equivalent. In such cases, the best that a reliable search algorithm can do is to return the entire Markov equivalence class, regardless of what features of the marginal density that it uses.

In contrast, for linear causal models with at most one error term is nonGaussian, SEMs *K* and *L* are Markov equivalent, but they are not distribution equivalent.

When Markov equivalence fails to entail distribution equivalence, using conditional independence relations alone for causal inference is still correct, but it is not as informative as theoretically possible. For example, assuming linearity, causal sufficiency, and nonGaussian errors (Shimizu et al. 2006), conditional independence tests can at best reliably determine the correct Markov equivalence class, while using other features of the sample density can be used to reliably determine a unique graph (Shimizu et al. 2006) or find information about latent variables. For example, linear graphical models entail rank constraints on various submatrices of the covariance matrix, regardless of the particular parameter values (Sullivant et al. 2010; Spirtes 2013). These rank constraints, together with conditional independence tests, can be used to identify models with latent confounders (Kummerfeld et al. 2014).

### Constraint-based search

The number of DAGs grows super-exponentially with the number of vertices, so even for modest numbers of variables, it is not possible to examine each DAG to determine whether it is compatible with the population density given the causal Markov and faithfulness assumptions. The PC algorithm, given as input an oracle that returns answers about conditional independence in the population and optional background knowledge about orientations of edges, returns a graphical object called a pattern that represents a Markov equivalence class (or if there is background knowledge a subset of a Markov equivalence class) on the basis of oracle queries. If the oracle always gives correct answers, and the causal Markov and causal faithfulness assumptions hold, then the output pattern contains the true SEM, even thought the algorithm does not check each DAG. In the worse case, it is exponential in the number of variables, but for sparse graphs, it can run on hundreds of thousands of variables (Spirtes and Glymour 1991; Spirtes et al. 1993; Meek 1995).

Recently, the general-purpose Boolean Satisfiability Solver (SAT), as a constrained optimization technique, has been used for causal discovery in a general model space (Hyttinen et al. 2013; Triantafillou and Tsamardinos 2015). Such methods make the use of conditional independence and dependence constraints and allow the integration of general background knowledge. They are able to discovery causal structures in the presence of both directed cycles (feedback loops) and latent variables from any given set of overlapping passive observational or experimental datasets. Since combinational optimization problems are essentially involved, such methods do not generally scale well as the number of variables increases.

## Differences between classification and regression and causal inference

The following is a brief summary of some important differences between the problem of predicting the value of a variable in an unmanipulated population from a sample and the problem of predicting the post-manipulation value of a variable from a sample from an unmanipulated population. In an unmanipulated population *P*, the predictor that minimizes the MSPE is the conditional expected value.

1. $E(Y|\mathbf {O})$ (the expected value of *Y* conditional on $\mathbf {O}$) is a function of *p*($\mathbf {O}$,*Y*), regardless of what the true causal model is.^6^ In contrast, a manipulated expected value is a function of $p(\mathbf {O},Y)$*and* a causal graph.
2. In order to determine whether $E_P(Y||p'(\mathbf {O}))$ (the expected value of *Y* after a manipulation to $p'(\mathbf {O})$) is a function of $p(\mathbf {O}, Y)$ and background knowledge, it is necessary to find *all* of the causal models compatible with $p(\mathbf {O}, Y)$ and background knowledge, not simply one causal model compatible with $p(\mathbf {O}, Y)$ and background knowledge.
3. Determining which causal models are compatible with background knowledge and a $p(\mathbf {O}, Y)$ requires making additional assumptions connecting population densities to causal models (e.g., causal Markov and faithfulness).
4. Without introducing some simplicity assumptions about causal models, for some common families of densities (e.g., Gaussian, multinomial), no $E_P(Y| \mathbf {O}'||p'(O))$ are functions of the population density without very strong background knowledge.
5. The justification for using simple statistical models is fundamentally different than the justification for using simple causal models. At a given sample size, the use of simple statistical model can be justified even if causal relations are not simple. However, the assumption that the simplest causal model compatible with $p(\mathbf {O}, Y)$ and background knowledge is a substantive assumption about the simplicity of mechanisms that exist in the world.
6. For many families of densities (e.g., Gaussian, multinomial), there is always a statistical model without hidden variables that contains the population density. For those same families of densities, a causal model that contains both the population probability density and the post-manipulation probability densities may require the introduction of unobserved variables.
7. Given a population density, and the set of causal models consistent with the population density and background knowledge, calculating the effects of a manipulation can be difficult because
  - There may be unobserved variables (even if only a single causal model is consistent with $p(\mathbf {O}, Y)$ and background knowledge).
  - There may be multiple causal models compatible with $p(\mathbf {O}, Y)$ and background knowledge.
8. For nonexperimental data, a post-manipulation density is different from the population density from which the sample is drawn. The post-manipulation values of the target variable *Y* are not directly measured in the sample. Hence, it is not possible to estimate the error in $E_P(Y| \mathbf {O}'||p'(O))$ by comparing it to the values in a sample from the $p(\mathbf {O}, Y)$.

## SEMs can help in causal discovery from I.I.D. and time series data

As discussed in "Constraint-Based search" section, the constraint-based approach to causal discovery involves conditional independence tests, which would be a difficult task if the form of dependence is unknown. It has the advantage that it is generally applicable, but the disadvantages are that faithfulness is a strong assumption and that it may require very large sample sizes to get good conditional independence tests. Furthermore, the solution of this approach to causal discovery is usually nonunique, and in particular, it does not help in determining causal direction in the two-variable case, where no conditional independence relationship is available.

What information can we use to fully determine the causal structure? A fundamental issue is, given two variables, how to distinguish cause from effect. To do so, one needs to find a way to capture the asymmetry between them. Intuitively, one may think that the physical process that generates effect from cause is more natural or simple in some way than recovering the cause from effect. How can we represent this generating process, and in which way is the causal process more natural or simple than the backward process?

Recently, several causal discovery approaches based on structural equation models (SEMs) have been proposed. A SEM represents the effect *Y* as a function of the direct causes *X* and some unmeasurable error:

1 $$\begin{aligned} Y = f(X, \varepsilon ; \varvec{\theta }_1), \end{aligned}$$

where $\varepsilon$ is the error term that is assumed to be independent from *X*, the function $f \in \mathcal {F}$ explains how *Y* is generated from *X*, $\mathcal {F}$ is an appropriately constrained functional class, and $\varvec{\theta }_1$ is the parameter set involved in *f*. We assume that the transformation from $(X,\varepsilon )$ to (*X*, *Y*) is invertible, such that *N* can be uniquely recovered from the observed variables *X* and *Y*.

For convenience of presentation, let us assume that both *X* and *Y* are one-dimensional variables. Without precise knowledge on the data-generating process, the SEM should be flexible enough such that it could be adapted to approximate the true data-generating process; more importantly, the causal direction implied by the SEM has to be identifiable in most cases, i.e., the model assumption, especially the independence between the error and cause, holds for only one direction, such that it implies the causal asymmetry between *X* and *Y*. Under the above conditions, one can then use SEMs to determine the causal direction between two variables, given that they have a direct causal relationship in between and do not have any confounder: for both directions, we fit the SEM, and then test for independence between the estimated error term and the hypothetical cause and the direction which gives an independent error term is considered plausible.

Several forms of the SEM have been shown to be able to produce unique causal directions and have received practical applications. In the linear, nonGaussian, and acyclic model [LiNGAM (Shimizu et al. 2006)], *f* is linear, and at most one of the error term $\varepsilon$ and cause *X* is Gaussian. The nonlinear additive noise model (Hoyer et al. 2009; Zhang and Hyvärinen 2009) assumes that *f* is nonlinear with additive noise (error) $\varepsilon$. In the post-nonlinear (PNL) causal model (Zhang and Hyvärinen 2009), the effect *Y* is further generated by a post-nonlinear transformation on the nonlinear effect of the cause *X* plus error term $\varepsilon$:

2 $$\begin{aligned} Y = f_2(f_1(X) + \varepsilon ), \end{aligned}$$

where both $f_1$ and $f_2$ are nonlinear functions and $f_2$ is assumed to be invertible.^7^ The post-nonlinear transformation $f_2$ represents the sensor or measurement distortion, which is frequently encountered in practice. In particular, the PNL causal model has a very general form [the former two are its special cases), but it has been shown to be identifiable in the generic case (except five specific situations given in (Zhang and Hyvärinen 2009)]. It is worth noting that it is not closed under marginalization, even if there are no confounders. In the subsequent sections, we will discuss the identifiability of various SEMs, how to distinguish cause from effect with the SEMs, and the relationships between different principles for causal discovery, including mutual independence of the error terms and the causal Markov condition, respectively.

Another issue we are concerned with is causal discovery from time series. According to Granger (1980), Granger’s causality in time series falls into the framework of constraint-based causal discovery combined with the temporal constraint that the effect cannot precede the cause. The SEM, together with the above temporal constraint, has also been exploited to estimate time-delayed causal relations possibly with instantaneous effects  (Zhang and Hyvärinen 2009). Compared to the conditional independence relationships, the SEM, if correctly specified, is able to recover more about the causal information. In this paper, when talking about causality in time series, we assume that the causal relations are linear with nonGaussian errors. In "Causal discovery from time series" section, after reviewing linear Granger causality with instantaneous effects, we focus on two problems which are traditionally difficult to solve. In particular, we present the theoretical results which make it possible to discover the temporal causal relations at the true causal frequency from subsampled data (Gong et al. 2015), that is, one can recover monthly causal relations from quarterly data or estimate rapid causal influences between stocks from their daily returns. Moreover, even when there exist confounder time series, theoretical results suggested that one can still identify the causal relations among the observed time series as well as the influences from the confounder series (Geiger et al. 2015).

## Several SEMs and the identifiability of causal direction

When talking about the causal relation between two variables, traditionally people were often concerned with the linear-Gaussian case, where the involved variables are Gaussian with a linear causal relation, or the discrete case. It turned out that the former case is one of the atypical situations where the causal asymmetry does not leave a footprint in the observed data or their joint distribution: the joint Gaussian distribution is fully determined by the mean and covariance, and with proper rescaling, the two variables are completely asymmetric w.r.t. the data distribution.

In the discrete case, if one knows precisely what SEM class generated the effect from cause, which, for instance, may be the noisy AND or noisy XOR gate, then under mild conditions, the causal direction can be easily seen from the data distribution. However, generally speaking, if the precise functional class of the causal process is unknown, in the discrete case it is difficult to recover the causal direction from observed data, especially when the cardinality of the variables is small. As an illustration, let us consider the situation where the causal process first generates continuous data and discretizes such data to produce the observed discrete ones. It is then not surprising that certain properties of the causal process are lost due to discretization, making causal discovery more difficult. In this paper we focus on the continuous case.

### Causal direction is not identifiable without constraints on SEMs

In the SEM (1), the error term is assumed to be independent from the cause. If for the reverse direction, one cannot find a function to represent *X* in terms of the hypothetical cause *Y* and an error term which is independent from *Y*, then we can determine the true causal direction or distinguish cause from effect. Unfortunately, this is not the case if we do not impose any constraint on the function *f*, as explained below.

According to Hyvärinen and Pajunen (1999), given *any* two random variables *X* and *Y* with continuous support, one can always construct another variable, denoted by $\tilde{\varepsilon }$, which is statically independent from *X*. In (Zhang et al. 2015) the class of functions to produce such an independent variable $\tilde{\varepsilon }$ (or called independent error term in our causal discovery context) was given, and it was shown that this procedure is invertible: *Y* is a function of *X* and $\tilde{\varepsilon }$.

This is also the case for the hypothetical causal direction $Y\rightarrow X$: we can also always represent *X* as a function of *Y* and an independent error term. That is, any two variables would be symmetric according to the SEM, if *f* is not constrained. Therefore, in order for the SEMs to be useful to determine the causal direction, we have to introduce certain constraints on the function *f* such that the independence condition on the error and the hypothetical cause holds for only one direction. Below we focus on the two-variable case, and the results can be readily extended to the case with an arbitrary number of variables, as shown in Peters et al. (2011).

### Linear non-Gaussian causal model

The linear causal model in the two-variable case can be written as

3 $$\begin{aligned} Y = bX + \varepsilon , \end{aligned}$$

where  Let us first give an illustration with simple examples why it is possible to identify the causal direction between two variables in the linear case. Assume *Y* is generated from *X* in a linear form, i.e., $Y = X + \varepsilon ,$ where 

**Fig. 3:**
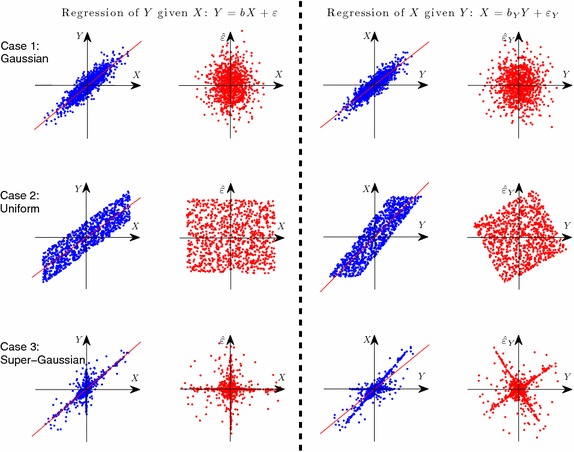
Illustration of causal asymmetry between two variables with linear relations. The data were generated according to equation 3 with , i.e., the causal relation is $X\rightarrow Y$. *From top to bottom*: *X* and $\varepsilon$ both follow the Gaussian distribution (case 1), uniform distribution (case 2), and a certain type of super-Gaussian distribution (case 3). The *two columns on the left* show the scatter plot of *X* and *Y* and that of *X* and the regression residual for regression of *Y* given *X*, and the *two columns on the right* correspond to regression of *X* given *Y*. Here we used 1000 data points. One can see that for regression of *X* given *Y*, in cases 2 and 3 the residual is not independent from the predictor, although they are uncorrelated by construction

 Figure 3 shows the scatterplot of 1000 data points of the two variables *X* and *Y* (columns 1 and 3) and that of the predictor and regression residual for two different regression tasks (columns 2 and 4). The three rows correspond to different settings: *X* and *E* are both Gaussian (case 1), uniformly distributed (case 2), and distributed according to some super-Gaussian distribution (case 3). In the latter two settings, *X* and *E* are nonGaussian, and one can see clearly that for regression of *X* given *Y* (the anti-causal or backward direction), the regression residual is not independent from the predictor any more. In other words, in those two situations, the regression residual is independent from the predictor only for the correct causal direction, giving rise to the causal asymmetry between *X* and *Y*.

Rigorously speaking, if at most one of *X* and $\varepsilon$ is Gaussian, the causal direction is identifiable, due to the independent component analysis (ICA) theory (Hyvärinen et al. 2001), or more fundamentally, due to the Darmois-Skitovich theorem (Kagan et al. 1973). This is known as the linear, nonGaussian, acyclic model [LiNGAM (Shimizu et al. 2006)]. Methods for estimating LiNGAM will be talked about in  "Determination of causal direction based on SEMs" section.

It is worth mentioning that in the linear case, it is possible to further estimate the effect of the underlying confounders in the system, if there are any, by exploiting overcomplete ICA (which allows more independent sources than observed variables) (Hoyer et al. 2008). Furthermore, when the underlying causal model has cycles or feedbacks, which violates the acyclicity assumption, one may still be able to reveal the causal knowledge under certain assumptions (Lacerda et al. 2008).

#### On the ubiquitousness of non-Gaussianity in the linear case

According to the central limit theorem, under mild conditions, the sum of independent variables tends to be Gaussian as the number of components becomes larger and larger. One may then challenge the nonGaussianity assumption in the LiNGAM model. Here we argue that in the linear case, nonGaussian distributions are ubiquitous.

Cramér’s decomposition theorem states that if the sum of two independent real-valued random variables is Gaussian, then both of the summand variables much be Gaussian as well; see [Cramér (1970), p. 53]. By induction, this means that if the sum of any finite independent real-valued variables is Gaussian, then all summands must be Gaussian. In other words, a Gaussian distribution can never be exactly produced by linear composition of variables any of which is nonGaussian. This nicely complements the central limit theorem: (under proper conditions) the sum of independent variable gets closer to Gaussian, but it cannot be exactly Gaussian, except that all summand variables are Gaussian. This linear closure property of the Gaussian distribution implies the rareness of the Gaussian distribution and ubiquitousness of nonGaussian distributions, if we believe the relations between variables are linear. However, the closer it gets to Gaussian, the harder it is to distinguish the direction. Hence, the practical question is, are the errors typically nonGaussian enough to distinguish causal directions in the linear case?

### Nonlinear additive noise model

In practice nonlinear transformation is often involved in the data-generating process and should be taken into account in the functional class. As a direct extension of LiNGAM, the nonlinear additive noise model represents the effect as a nonlinear function of the cause plus independent error (Hoyer et al. 2009):

4 $$\begin{aligned} Y = f_{AN}(X) + \varepsilon . \end{aligned}$$

It has been shown that the set of all *p*(*X*) for which the backward model also admits an independent error term is contained in a 3-dimensional affine space. Bearing in mind that the space of all possible *p*(*X*) is infinite dimensional, one can see that roughly speaking, in the generic case, if the data were generated by the nonlinear additive noise model, the causal direction is identifiable. This model is a special case of the PNL causal model, which is to be discussed below, and the identifiability results for the PNL causal model also apply here.

With certain modifications, the additive noise model also applies to discrete variables to represent a certain type of data-generating process in the discrete case (Peters et al. 2010). The additive noise model has also been used to model cyclic causal relations between two variables at an equilibrium state (Mooij et al. 2011).

### Post-nonlinear causal model

If the assumed SEM is too restrictive to be able to approximate the true data-generating process, the causal discovery results may be misleading. Therefore, if the specific knowledge about the data-generating mechanism is not available, to make it useful in practice, the assumed causal model should be general enough, such that it can reveal the data-generating processes approximately.

The PNL causal model takes into account the nonlinear influence from the cause, the noise effect, and the possible sensor or measurement distortion in the observed variables (Zhang and Hyvärinen 2009, 2010). See Eq. (2) for its form; a slightly more restricted version of the model, in which the inner function, $f_1$, is also assumed to be invertible, and was proposed in Zhang and Chan (2006) and applied to causal analysis of stock returns. It has the most general form among all well-defined SEMs according to which the causal direction is identifiable in the general case. (The model used in Mooij et al. (2010) does not impose structural constraints but assumes a certain type of smoothness; however, it does not lead to theoretical identifiability results.) Clearly it contains the linear model and nonlinear additive noise model as special cases. The multiplicative noise model, $Y=X\cdot \varepsilon$, where all involved variables are positive, is another special case, since it can be written as $Y= \exp ({\log X + \log \varepsilon })$, where $\log \varepsilon$ is considered as a new noise term, $f_1(X) = \log (X)$, and $f_2(\cdot ) = \exp (\cdot )$.

#### Theoretical identifiability of the causal direction

As stated in  "Causal direction is not identifiable without constraints on SEMs" section, the identifiability of the causal direction is a crucial issue in SEM-based causal discovery. Since LiNGAM and the nonlinear additive noise model are special cases of the PNL causal model, the identifiability conditions of the causal direction for the PNL causal model also entail those for the former two SEMs.

Such identifiability conditions for the PNL causal model were established by a proof by contradiction (Zhang and Hyvärinen 2009). We assume the causal model holds in both directions $X\rightarrow Y$ and $Y\rightarrow X$, and show that this implies very strong conditions on the distributions and functions involved in the model. Suppose the data were generated according to the PNL causal model in settings other than those specific conditions; then in principle, the backward direction does not follow the model, and the causal direction can be determined.

Assume that the data (*X*, *Y*) are generated by the PNL causal model with the causal relation $X \rightarrow Y$. This data-generating process can be described as (2). Moreover, let us assume that the backward direction, $Y \rightarrow X$ also follows the PNL causal model with independent error. That is,

5 $$\begin{aligned} X = g_2(g_1(Y) + \varepsilon _Y), \end{aligned}$$

where *Y* and $\varepsilon _Y$ are independent, $g_1$ is nonconstant, and $g_2$ is invertible.

Equations (2) and (5) define the transformation from $(X, \varepsilon )^\intercal$ to $(Y, \varepsilon _Y)^\intercal$; as a consequence, $p(Y, \varepsilon _Y)$ can be expressed in terms of $p(X, \varepsilon ) = p(X) p(\varepsilon )$. The identifiability results were obtained based on the linear separability of the logarithm of the joint density of independent variables, i.e., for a set of independent random variables whose joint density is twice differentiable, the Hessian of the logarithm of their density is diagonal everywhere (Lin 1998). Since *Y* and $\varepsilon _Y$ are assumed to be independent, $\log p(Y, \varepsilon _Y)$ then follows such a linear separability property. This implies that the second-order partial derivative of $\log p(Y, \varepsilon _Y)$ w.r.t. *Y* and $\varepsilon _Y$ is zero. It then reduces to a differential equation of a bilinear form. Under certain conditions (e.g., $p(\varepsilon )$ is positive on $(-\infty , +\infty )$), the solution to the differential equation gives all cases in which the causal direction is *not* identifiable according to the PNL causal model. Table 1 in Zhang and Hyvärinen (2009) summarizes all five nonidentifiable cases. The first one is the linear-Gaussian case, in which the causal direction is well known to be nonidentifiable. Roughly speaking, to make one of those cases true, one has to adjust the data distribution and the involved nonlinear functions very carefully. In other words, in the generic case, the causal direction is identifiable if the data were generated according to the PNL causal model.

### Nonlinear deterministic case: information-geometric causal inference

Suppose *Y* was generated from *X* by a nonlinear deterministic and invertible function, i.e., $Y = h(X)$; is it possible to distinguish cause from effect? One way to tackle this problem is to make use of a certain type of independence between *p*(*X*) and the transformation *h* (Daniusis et al. 2010; Janzing et al. 2012). In particular, they considered *p*(*X*) and $\log |h'(X)|$ as random processes indexed by *x* values and showed that if they are uncorrelated w.r.t. a reference measure (e.g., the uniform distribution), then for the reverse direction, *p*(*Y*) and $\log |(h^{-1})'(Y)|$ are positively correlated, implying the asymmetry between *X* and *Y*. Based on this observation, the methods of information-geometric causal inference (IGCI) was derived.

In this case, the identifiability of the causal direction relies on the assumption that the causal process is noiseless. Moreover, IGCI assumes that the distributions *p*(*X*) and *p*(*Y*) and the log-derivative of the nonlinear transformation, $\log |h'(X)|$, are complex enough so that one can assess the correlation and compare the two candidate directions reliably.

## Determination of causal direction based on SEMs

LiNGAM can be estimated from observational data in a computationally relatively efficient way. Suppose we aim to estimate the causal model underlying the observable random vector $\mathbf {X}=(X_1,...,X_n)^\intercal$. In matrix form we can represent such causal relations with a matrix $\mathbf {B}$, i.e., $\mathbf {X} = \mathbf {BX} + \mathbf {E}$, where $\mathbf {B}$ can be permuted to a strictly lower-triangular matrix and $\mathbf {E}$ is the vector of independent error terms. This can be rewritten as

6 $$\begin{aligned} \mathbf {E} = (\mathbf {I}-\mathbf {B})\mathbf {X}, \end{aligned}$$

where $\mathbf {I}$ denotes the identity matrix. The approach of ICA-LiNGAM (Shimizu et al. 2006) estimates the matrix $\mathbf {B}$ in two steps. It first applies ICA (Hyvärinen et al. 2001) on the data:

7 $$\begin{aligned} \mathbf {Z} = \mathbf {WX}, \end{aligned}$$

such that $\mathbf {Z}$ has independent components. Second, an estimate of $\mathbf {B}$ can be found by permuting and rescaling the matrix $\mathbf {W}$, as implied by the correspondence between Eqs. 6 and 7.

As the number of variables, *n*, increases, the estimated linear transformation $\mathbf {W}$ may converge to local optima more likely and involve more and more random errors, causing estimation errors in the causal model. Bear in mind that the causal matrix we aim to estimate, $\mathbf {B}$, is very sparse because it can be permuted to a strictly lower-triangular matrix. Hence, to improve the estimation efficiency, one may enforce the sparsity constraint on the entries of $\mathbf {W}$, as achieved by ICA with sparse connections (Zhang et al. 2009). Another way to reduce the estimation error is to find the causal ordering by recursively performing regression and independence test between the predictor and residual, as done by DirectLiNGAM (Shimizu et al. 2011).

However, generally speaking, causal discovery based on nonlinear SEMs are not computationally as efficient as in the linear case. A commonly used approach to distinguishing cause from effect with nonlinear SEMs consists of two steps. First, one fits the model (e.g., the nonlinear additive noise model or the PNL causal model) on the data for both hypothetical causal directions. The second step is to do independence test between the estimated error term and hypothetical cause (Hoyer et al. 2009; Zhang and Hyvärinen 2009). If the independence condition holds for one and only one hypothetical direction, the causal relation between the two variables *X* and *Y* implied by the corresponding SEM has been successfully found. If neither of them holds, the data-generating process may not follow the assumed SEM, or there exists some confounder influencing both *X* and *Y*. If both hold, the cause and effect cannot be distinguished by the exploited SEM; in this case, additional information, such as the smoothness of the involved nonlinearities, may help find the causal model with a lower complexity. We adopted the Hilbert Schmidt information criterion (HSIC) (Gretton et al. 2005) for statistical independence test in the first step. Below we discuss how to estimate the function as well as the error term in the first step.

For the nonlinear additive noise model, the function $f_{AN}$ is usually estimated by performing Gaussian process (GP) regression (Hoyer et al. 2009). For details on GP regression, one may refer to Rasmussen and Williams (2006).

Estimation of the PNL causal model (2) has several indeterminacies: the sign, mean, and scale of the error term *varepsilon*, and accordingly, the sign, location, and scale of $f_{i1}$ are arbitrary. In the estimation procedure, one may impose certain constraints to avoid such indeterminacies in the estimate. However, we should note that in principle, we do not care about those indeterminacies in the causal discovery context, since they do not change the statistical independence or dependence property between the estimated error term and the hypothetical cause.

It is well known that for linear regression, the maximum likelihood estimator of the coefficient is still statistically consistent even if the error distribution is wrongly assumed to the Gaussian. However, this may not be the case for general nonlinear models. As shown in [Zhang et al. (2015), Section 3.2], if the error distribution mis-specified, the estimated PNL causal model (2) may not be statistically consistent, even when the above indeterminacies in the estimate are properly tackled. Therefore, the error distribution should be adaptively estimated from data, if the true one is not known *a priori*. It has been proposed to estimate the PNL causal model (2) by mutual information minimization (Zhang and Hyvärinen 2009) with the involved nonlinear functions represented by multi-layer perceptrons (MLPs). Later, in Zhang et al. (2015) the PNL causal model was estimated by extending the framework of warped Gaussian processes to allow a flexible error distribution, which is represented by a mixture of Gaussians (MoG).

## On the relationships among different principles for model estimation

One usually uses maximum likelihood to fit the SEM together with a DAG to the given data. Not surprisingly, the negative likelihood (with the distribution of the error term adaptively estimated from data) is equivalent to the mutual information between the estimated error terms, as stated in Theorem 3 in Zhang et al. (2015). The higher the likelihood, the less dependent the estimated error terms. (Note that the root variables in the DAG are also counted as error terms.)

On the other hand, the constraint-based approach to causal discovery exploits conditional independence relationships of the variables to derive (the equivalence class of) the causal structure (Spirtes et al. 2001; Pearl 2000). How are these principles, including mutual independence of the estimated error terms and the causal Markov condition, related to each other? Below we will answer this question, and the results in this section hold for an arbitrary number of variables.

Let us consider optimization over different DAG structures to find the causal structure. Assume that we optimally fit the nonlinear functions $f_i$ according to the given candidate DAG structure. First consider the situation where we fit the nonlinear additive noise model, i.e.,

8 $$\begin{aligned} X_i = f_{AN,i}(\mathbf {PA}_i) + \varepsilon _i, \end{aligned}$$

to the data. It has been shown that mutual independence of the error terms and conditional independence between observed variables (together with the independence between $\varepsilon _i$ and $\mathbf {PA}_i$) are equivalent. Furthermore, they are achieved if and only if the total entropy of the disturbances is minimized (Zhang and Hyvärinen 2009). More specifically, when fitting the model (8) with a hypothetical DAG causal structure to the given variables $X_1,\ldots , X_n$, the following three properties are equivalent:

1. The causal Markov condition holds (i.e., each variable is independent of its nondescendants in the DAG conditioning on its parents), and in addition, the error term in $X_i$ is independent from the parents of $X_i$.
2. The error terms $N_i$ are mutually independent.
3. The total entropy of the error terms, i.e., $\sum _{i}H(\varepsilon _i)$, is minimized, with the minimum $H(X_1,\ldots , X_n)$.

Let us then consider the PNL causal model. When one fits the PNL causal model

9 $$\begin{aligned} X_i = f_{i2}(f_{i1}(\mathbf {PA}_i) + \varepsilon _i), \end{aligned}$$

to the data, the scale of the error terms as well as $f_{i1}$ is arbitrary, since $f_{i2}$ is also to be estimated. Consequently, unlike for the nonlinear additive noise model, in the PNL causal model context, it is not meaningful to talk about the total entropy of the error terms (see condition (3) above). However, as shown in Zhang and Hyvärinen (2009), when fitting the PNL causal model with a hypothetical DAG causal structure to the data, we still have the equivalence between conditions (1) and (2) above.

Given more than two variables, one way to estimate the causal model based on SEMs is to use exhaustive search: for all possible causal orderings, fit SEMs for all hypothetical effects separately, and then do model checking by testing for independence between the estimated error and the corresponding hypothetical causes. However, note that the complexity of this procedure increases super-exponentially along with the number of variables. Smart approaches are then needed.

The above result concerning the relationship between mutual independence of the error terms and the causal Markov condition combined with the independence between each error term, and its associated parents suggests a two-step method to find the causal structure implied by the PNL causal model. One first uses the constraint-based approach to find the Markov equivalent class from conditional independence relationships with proper nonparametric conditional independence tests (e.g., Zhang et al. (2011)). The PNL causal model is then used to identify the causal directions that cannot be determined in the first step: for each DAG contained in the equivalent class, we estimate the error terms and determine whether this causal structure is plausible by examining whether the disturbance in each variable $X_i$ is independent from the parents of $X_i$. Consequently, one avoids the exhaustive search over all possible causal structures and high-dimensional statistical tests of mutual independence of all error terms. In the context of nonlinear additive noise model, such a hybrid scheme for causal discovery of more than two variables has been discussed in Zhang and Hyvärinen (2009), Tillman et al. (2009).

## Causal discovery from time series

Both the constraint-based and SEM-based approaches to causal discovery are directly applicable to find causal relations over the random variables involved in the stochastic processes (or time series); moreover, one can benefit from the temporal constraint that the effect cannot precede the cause, which helps reduce the search space of the causal structure. The work Eichler (2012) provides an overview over various definitions of causation w.r.t. time series and reviews some causal discovery methods. Below we mainly consider SEM-based causal discovery from time series; more specifically, we assume linearity of the causal relations and consider three problems, namely linear Granger causal analysis with instantaneous effects, causal discovery from systematically subsampled data, and that in the presence of hidden time series.

### Linear Granger causality and its extension with instantaneous effects

For Granger causal analysis in the linear case Granger (1980), one fits the following VAR model (Sims 1980) to the data:

10 $$\begin{aligned} \mathbf {X}_t = \mathbf {A} \mathbf {X}_{t-1} + \varvec{\varepsilon }_t, \end{aligned}$$

where $\mathbf {X}_t = (X_{1t}, X_{2t}, ..., X_{nt})^\intercal$ is the vector of the observed data, $\varvec{\varepsilon }_t = (\varepsilon _{1t}, ..., \varepsilon _{nt})^\intercal$ is the temporally and contemporaneously independent noise process, and causal transition matrix $\mathbf {A}$ contains the temporal causal relations.

In practice it is found that after fitting the VAR model, the residuals are often contemporaneously dependent. To account for such dependence, the above VAR model has been extended to allow instantaneous causal effects between $X_{it}$ (Hyvärinen et al. 2010). Let $\mathbf {B}_0$ contains the instantaneous causal relations between $\mathbf {X}_t$. Equation (10) changes to

11 $$\begin{aligned}&\mathbf {X}_t = \mathbf {B}_0 \mathbf {X}_t + \mathbf {A} \mathbf {X}_{t-1} + \varvec{\varepsilon }_t, \nonumber \\ \Rightarrow&(\mathbf {I} - \mathbf {B}_0)\mathbf {X}_t = \mathbf {A} \mathbf {X}_{t-1} + \varvec{\varepsilon }_t ,\nonumber \\ \Rightarrow&\mathbf {X}_t = (\mathbf {I} - \mathbf {B}_0)^{-1}\mathbf {A} \mathbf {X}_{t-1} + (\mathbf {I} - \mathbf {B}_0)^{-1} \varvec{\varepsilon }_t. \end{aligned}$$

To estimate all involved parameters in Granger causality with instantaneous effects, two estimation procedures have been proposed in Hyvärinen et al. (2010). The two-step method first estimates the errors in the above VAR model and then applies independent component analysis (ICA) (Hyvärinen et al. 2001) on the estimated errors. The other is based on multichannel blind deconvolution, which is statistically more efficient (Zhang and Hyvärinen 2009).

### Causal discovery from subsampled data

Suppose the original high-resolution data were generated by (10). We consider low-resolution data generated by subsampling (or systematic sampling) with the subsampling factor *k*. The work (Danks and Plis 2014) aims to infer the causal structure at the correct causal frequency directly from the causal structure learned from the subsampled data; they do not assume any specific form for the causal relations, and their method is completely nonparametric, but on the other hand, an MCMC search is needed, which involves high computational load, and this method cannot estimate the strength of the causal relations.

Alternatively, one may assume an SEM for the underlying causal model at the true causal frequency, which may be fully identifiable from subsampled data. In particular, let us consider the linear case; one is then interested in finding the causal transition matrix $\mathbf {A}$ at the true causal frequency. Traditionally, if one uses only the second-order information, this suffers from parameter identification issues (Palm and Nijman 1984), i.e., the same subsampled (low-frequency) model may disaggregate to several high frequency models, which are observationally equivalent at the low frequency.

#### Effect of subsampling (systematic sampling)

Suppose that due to low resolution of the data, there is an observation every *k* time steps. That is, the low-resolution observations $\tilde{\mathbf {X}} = (\tilde{\mathbf {X}}_1, \tilde{\mathbf {X}}_2,, ...,\tilde{\mathbf {X}}_t)$ are $(\mathbf {X}_{1}, \mathbf {X}_{1+k}, ..., \mathbf {X}_{1+(t-1)k} )$; here we have assumed that the first sampled point is $\mathbf {Xx}_1$. We then have

12 $$\begin{aligned} \tilde{\mathbf {X}}_{t+1}&= \mathbf {X}_{1+tk} = \mathbf {A}\mathbf {X}_{1+tk-1} + \varvec{\varepsilon }_{1+tk} \nonumber \\&= \mathbf {A}(\mathbf {A}\mathbf {X}_{1+tk-2} + \varvec{\varepsilon }_{1+tk-1}) + \varvec{\varepsilon }_{1+tk} \nonumber \\&=...\nonumber \\&= \mathbf {A}^k \tilde{\mathbf {X}}_t + \underbrace{\sum _{l=0}^{k-1} \mathbf {A}^{l} \varvec{\varepsilon }_{1+tk-l}}_{\triangleq \mathbf {\varvec{\varepsilon }}_t}. \end{aligned}$$

According to (12), subsampled data $\tilde{\mathbf {X}}_t$ also follows a vector autoregression (VAR) model with the error term $\mathbf {\varvec{\varepsilon }}_t$, and one can see that as $T\rightarrow \infty$, the discovered temporal causal relations from such subsampled data are given by $\mathbf {A}^k$. As $k\rightarrow \infty$, $\mathbf {A}^k$ tends to vanish, and the subsampled data will be contemporaneously dependent. (We have assumed that the system is stable, in that all eigenvalues of $\mathbf {A}$ have modulus smaller than one.)

#### Misleading Granger causal relations in low-resolution data 

An illustration Suppose $$A = \begin{bmatrix} 0.8&0.5 \\ 0&-0.8 \end{bmatrix}$$. Consider the case where $k=2$. The corresponding VAR model for the subsampled data is

$$\begin{aligned} \tilde{\mathbf {X}}_t = \mathbf {A}^2 \tilde{\mathbf {X}}_{t-1} + \mathbf {\varvec{\varepsilon }}_{t} = \begin{bmatrix} 0.64 &\quad 0 \\ 0 &\quad 0.64 \end{bmatrix} \tilde{\mathbf {X}}_{t-1} + \mathbf {\varvec{\varepsilon }}_{t} . \end{aligned}$$

That is, the causal influence from ${X}_{2,t-1}$ to ${X}_{1t}$ is missing in the corresponding low-resolution data (with $k=2$).

#### Identifiability of the causal relations at the causal frequency

It has been shown that if the distributions $p_{N_i}$ are nonGaussian and different for different *i*, together with other technical assumptions, the transition matrix associated with the causal-frequency data, $\mathbf {A}$, is identifiable from the subsampled data $\tilde{\mathbf {X}}$. As a by-product, the result also indicates that the subsampled data, although contemporaneously dependent, actually *do not* follow the model of linear Granger causality with instantaneous effects (Gong et al. 2015).

Let the distributions of the noise terms be represented by the MoG. An EM algorithm and a variational EM (with mean field approximation) were then proposed to estimate $\mathbf {A}$ from subsampled data.

### Causal discovery with hidden time series (Confounders)

In practice it is usually difficult and even impossible to collect all relevant time series when doing causal analysis on given ones. We approach this problem as follows: We assume that the (multivariate) measurements are a sample of a multivariate random process $\mathbf {X}_t$, which, together with another random process $\mathbf {Z}_t$, forms a VAR process. That is,

13 $$\begin{aligned} \begin{bmatrix} \mathbf {X}_t \\ \mathbf {Z}_t \end{bmatrix} = \begin{bmatrix} \mathbf {B}&\mathbf {C} \\ \mathbf {D}&\mathbf {E} \end{bmatrix} \cdot \begin{bmatrix} \mathbf {X}_{t-1} \\ \mathbf {Z}_{t-1} \end{bmatrix} + {\varvec{\varepsilon }}_t, \end{aligned}$$

where $\mathbf {Z}_t$ is not measured and can be considered as confounder time series, $\mathbf {B}$ is the causal transition matrix for the observed process $\mathbf {X}_t$, and $\mathbf {C}$ contains the influence from $\mathbf {Z}_t$ to the observed process $\mathbf {X}_t$. The theoretical issue is whether $\mathbf {B}$ and $\mathbf {C}$ are identifiable from solely the observed process $\mathbf {X}_t$.

#### Practical Granger causal analysis can go wrong

In practical Granger causal analysis, one just performs a linear regression of present on past on the observed $\mathbf {X}_t$ and then interprets the regression matrix causally. While making the ideal definition practically feasible, this may lead to wrong causal conclusions in the sense that it does not comply with the causal structure that we would infer, given we had more information. Let us give an example for this. Let $\mathbf {X}_t$ be bivariate and $\mathbf {Z}_t$ be univariate. Moreover, assume

$$\begin{aligned} \begin{bmatrix} \mathbf {B}&\mathbf {C} \\ \mathbf {D}&\mathbf {E} \end{bmatrix} = \left( \begin{array}{cccc} 0.9 &{} 0 &{}&{} 0.5 \\ 0.1 &{} 0.1 &{}&{} 0.8 \\ \hline 0 &{} 0 &{}&{} 0.9 \\ \end{array} \right) , \end{aligned}$$

and let the covariance matrix of ${\varvec{\varepsilon }}_t$ be the identity matrix. To perform practical Granger causal analysis, we proceed as usual: we fit a VAR model on *only* the observable process $\mathbf {X}_t$, in particular calculate the VAR transition matrix by

$$\begin{aligned} B_{pG} = \mathbb {E}(\mathbf {X}_t \mathbf {X}_{t-1}^\intercal )\mathbb {E}^{-1}(\mathbf {X}_t \mathbf {X}_t^\intercal ) = \left( \begin{array}{cc} 0.89 & \quad {} 0.35\\ 0.08 & \quad {} 0.65\\ \end{array} \right) . \end{aligned}$$

(up to rounding), and interpret the coefficients of $B_{pG}$ as causal influences. Although, according to $\mathbf {B}$, the true time-delayed causal relations in $\mathbf {X}_t$, $X_{2t}$ does not cause $X_{1t}$, $B_{pG}$ suggests that there is a strong causal effect $X_{2,t-1} \rightarrow X_{1t}$ with the strength 0.35. It is even stronger than the relation $X_{1,t-1} \rightarrow X_{2t}$, which actually exists in the complete model with the strength 0.1.

#### Identifiability of $\mathbf {B}$ and Almost Identifiability of $\mathbf {C}$

Assume that all components of ${\varvec{\varepsilon }}_t$ are nonGaussian and that the dimensionality of the hidden process $\mathbf {Z}_t$ is not higher than that of the observed process $\mathbf {X}_t$. Together with some further technical assumptions, it has been shown that $\mathbf {B}$ is identifiable from $\mathbf {X}_t$; furthermore, the set of columns of $\mathbf {C}$ with at least two nonzero entries is identifiable from up to scaling of those columns (Geiger et al. 2015).

One can then use a MoG to represent the distributions of the components of ${\varvec{\varepsilon }}_t$ and develop a variation EM algorithm to estimate $\mathbf {B}$ and $\mathbf {C}$ from solely $\mathbf {X}_t$.

## Conclusion and open problems

We have reviewed central concepts in and fundamental methodologies for causal inference and discovery. The concepts include manipulations, causal models, sample predictive modeling, causal predictive modeling, structural equation models, the causal Markov assumption, and the faithfulness assumption. We have discussed the constraint-based causal structure search and its properties. In the second part of the paper, we have given a survey of structural equation models which enable us to fully identify causal structure from observational data. We focused on the two-variable case, where the task is to distinguish cause from effect. We have reviewed the linear nonGaussian causal model, nonlinear additive noise model, and the post-nonlinear causal model, listed from the most to the least restrictive. We addressed the identifiability of the causal direction: for those three models, in the generic case, the backward direction does not admit an independent error term, and, as a consequence, it is possible to distinguish cause from effect. We have also briefly discussed the procedure to do so, which consists of fitting the structural equation model and doing independence test between the estimated error term and the hypothetical cause.

In the last three decades, enlightening progress has been made in the field of causal discovery and inference. However, there are still many fundamental questions to be answered:^8^

- What new models are appropriate for different combinations of kinds of data, e.g., experimental and observational (Cooper and Yoo 1999; Danks 2002; Yoo and Cooper 2004; Eberhardt et al. 2005; Yoo et al. 2006; Eberhardt et al. 2006)?
- What new models are appropriate for different kinds of background knowledge, and different families of densities?
- What kind of scores can be used to best evaluate causal models from various kinds of data? In a related vein, what are good families of prior distributions that capture various kinds of background knowledge?
- How can search algorithms be improved to incorporate different kinds of background knowledge, search over different classes of causal models, run faster, handle more variables and larger sample sizes, be more reliable at small sample sizes, and produced output that is as informative as possible?
- For existing and novel causal search algorithms, what are their semantic and syntactic properties (e.g., soundness, consistency, maximum informativeness)? What are their statistical properties (pointwise consistency, uniform consistency, sample efficiency)? What are their computational properties (computational complexity)?
- What plausible alternatives are there to the Causal Markov and Faithfulness Assumptions? Are there other assumptions might be weaker and hold in more domains and applications without much loss about what can be reliably inferred? Are there stronger assumptions that are plausible for some domains that might allow for stronger causal inferences? How often are these assumptions violated, and how much do violations of these assumptions lead to incorrect inferences?
- There are special assumptions, such as linearity, which can improve the strength of causal conclusions that can be reliably inferred, and the speed and sample efficiency of algorithms that draw the conclusions. What other distribution families or stronger assumptions about a domain are there that are plausible for some domains and how can they be used to improve causal inference?
- Can various statistical assumptions be relaxed? For example, what if the sample selection process is not i.i.d., but may be causally affected by variables of interest (Cooper 1995; Spirtes et al. 1995; Cox and Wermuth 1996; Cooper 2000; Richardson and Spirtes 2002)?

In addition, there are also a number of open problems concerning SEM-based causal discovery and the asymmetry between cause and effect.

- First, one can consider structural equation models as a way to represent the conditional distribution of the effect given the cause. Can we then find hints as to the causal direction directly from the data distribution? In other words, can we find a general way to directly characterize the causal asymmetry in light of certain properties of the data distribution? If we managed to do so, it would hopefully put the causal Markov condition, the independent noise condition (in the SEMs), and the independent transformation condition in the nonlinear noiseless case (Janzing et al. 2012) under the same umbrella. To this end, an attempt has been made by exploiting the so-called “exogeneity” property of a causally sufficient causal system (Zhang et al. 2015). But it is not clear whether this property is able to bring about computationally efficient and widely applicable causal discovery methods. Like the work Mooij et al. (2010), it might be difficult or even impossible to derive theoretical identifiability conditions of the causal direction for such a method.
- Secondly, note that nonlinear structural equation models are usually intransitive. That is, if both causal processes $X_1 \rightarrow X_2$ and $X_2 \rightarrow X_3$ admit a particular type of structural equation model, say, the nonlinear additive noise model, the process $X_1 \rightarrow X_3$ does not necessarily follow the same model. (Linear models are transitive.) This could be a potential issue with structural equation model-based causal discovery: it may fail to discover indirect causal relations. (Here by direction causal relations, we mean the causal relations in which only a single-noise variable is involved.) On the other hand, this may be a benefit of using structural equation models for causal discovery, in that it is possible to detect the existence of causal intermediate variables and further recover them. But how to do so is currently unclear.
- We have discussed how different types of independence, including conditional independence in the causal Markov condition and statistical independence between the error term and hypothetical cause in structural equations models, help discovery causal information from data. On the other hand, it has been demonstrated that this type of independence (which is, loosely speaking, the independence between how the cause is generated and how the effect is generated from cause) is able to facilitate understanding and solving some machine learning or data analysis problems. For instance, it implies that when the feature causes the label (or target), unlabeled data points will *not* help in the semi-supervised learning scenario (Schölkopf et al. 2012), and inspired new settings and formulations for domain adaptation by characterizing what information to transfer (Zhang et al. 2013, 2015). It is under investigation whether other machine learning methods including “adaptive boosting” can be understood from the causal perspective. In addition, it is unclear whether the learning guarantees for supervised learning actually depend on the causal relationship between the feature and target (or label), i.e., the causal role of the feature w.r.t. the target.
- Next, developing efficient methods for causal discovery of more than two variables based on structural equation models is an important step towards large-scale causal analysis in various domains including neuroscience and biology. To make causal discovery computationally efficient, one may have to limit the complexity of the causal structure, say, limit the number of direct causes of each variable. Even so, a smart optimization procedure instead of exhaustive search is still missing in the literature.
- Finally, in causal analysis of large-scale real-world systems, there are usually many practical issues to consider. For instance, unmeasured confounders usually cause much difficulty in causal discovery, and one may combine the FCI algorithm (Spirtes et al. 1995), which is a constraint-based method allowing confounders, with appropriate methods for SEM-based causal discovery. Because an undirected graph that represents a probability distribution *p* contains a superset of the adjacencies in a pattern that represents *p*, which in turn contains a superset of the adjacencies in a PAG that represents *p*, the output of an undirected graph search or a pattern search can be used as the starting point of a constraint-based search for a PAG, instead of starting with a complete undirected graph as the starting point (as FCI currently does). But an optimal way to do so is to be explored. Moreover, in practice, especially in finance, economics, and neuroscience, the causal model may be time-varying. There exist some methods aiming to detect the changes [Talih and Hengartner (2005); Adams and Mackay 2007); Kummerfeld and Danks 2013)] or directly model time-varying causal relations (see, e.g., Huang et al. (2015)) in a dynamic manner. They usually focus on the linear case and cannot quickly locate changing causal relations. The work (Zhang et al. 2015) extends constraint-based causal discovery to be able to directly determine those variables with changing generating processes and discover the correct causal skeleton. However, it does not show how the causal relations change over time. It is of practical importance to develop methods they are able to detect and estimate time-varying causal models efficiently (in both statistical and computational senses).

### Software packages and source code

The following software packages are available online:

- The Tetrad project webpage (Tetrad implements a large number of causal discovery methods, including PC and its variants, FCI, and LiNGAM): http://www.phil.cmu.edu/tetrad/.
- Kernel-based conditional independence test Zhang et al. (2011): http://people.tuebingen.mpg.de/kzhang/KCI-test.zip.
- LiNGAM and its extensions Shimizu et al. (2006, 2011): https://sites.google.com/site/sshimizu06/lingam.
- Fitting the nonlinear additive noise model Hoyer et al. (2009): http://webdav.tuebingen.mpg.de/causality/additive-noise.tar.gz.
- Distinguishing cause from effect based on the PNL causal model Zhang and Hyvärinen (2009, 2010): http://webdav.tuebingen.mpg.de/causality/CauseOrEffect_NICA.rar.
- Probabilistic latent variable models for distinguishing between cause and effect Mooij et al. (2010): http://webdav.tuebingen.mpg.de/causality/nips2010-gpi-code.tar.gz.
- Information-geometric causal inference Daniusis et al. (2010); Janzing et al. (2012): http://webdav.tuebingen.mpg.de/causality/igci.tar.gz.

## Footnotes

1 Here, the “population” is simply a collection of instantiations of a set of random variables. For example, it could consist of a set of satellite readings and rainfall rates in different locations at a given time, or the readings of a single satellite and rainfall rate over time, or a combination of these.

2 In order to avoid technicalities, we will assume that the set of values conditioned on do not have measure 0.

3 Other measures of prediction error, such as the absolute value of prediction error or optimizing certain decision problems, could be used but would not substantially change the general approach taken here.

4 Here, $p'$ is not a derivative of *p*; the prime after the *p* merely indicates that a new function has been introduced. The use of manipulated probability densities in decision theory is often not explicit. The assumption that the density of states of nature is independent of the actions taken (act-state independence) is one way to ensure that the manipulated densities that are needed are equal to observed conditional densities that can be measured.

5 A smooth measure is absolutely continuous with Lebesgue measure.

6 This ignores the problem of conditioning on sets of measure zero.

7 In (Zhang and Chan 2006) both functions $f_1$ and $f_2$ are assumed to invertible; this causal model, as a consequence, can be estimated by making use of post-nonlinear independent component analysis (PNL-ICA) Taleb and Jutten (1999), which assumes that the observed data are component-wise invertible transformations of linear mixtures of the independence sources to be recovered.

8 The content and organization of the following open questions are largely due to suggestions from Constantin Aliferis, whom we thank for his suggestions.
